# The Dark Annulus of a Drop in a Hele-Shaw Cell Is Caused by the Refraction of Light through Its Meniscus

**DOI:** 10.3390/mi13071021

**Published:** 2022-06-28

**Authors:** Sangjin Ryu, Haipeng Zhang, Carson Emeigh

**Affiliations:** 1 Department of Mechanical and Materials Engineering, University of Nebraska-Lincoln, Lincoln, NE 68588, USA; haipengz@uic.edu (H.Z.); carsonemeigh0521@gmail.com (C.E.); 2 Nebraska Center for Materials and Nanoscience, University of Nebraska-Lincoln, Lincoln, NE 68588, USA

**Keywords:** confocal fluorescence microscopy, 3D imaging, microchannel, image processing, surface tension coefficient, refractive index, ray optics, micro optofluidics

## Abstract

Knowing the meniscus shape of confined drops is important for understanding how they make first contact and then coalesce. When imaged from the top view by brightfield microscopy, a liquid drop (e.g., corn syrup) confined in a Hele-Shaw cell, surrounded by immiscible liquid (e.g., mineral oil), had a dark annulus, and the width of the annulus decreased with increasing concentration of corn syrup. Since the difference in the annulus width was presumed to be related to the meniscus shape of the drops, three-dimensional images of the drops with different concentrations were obtained using confocal fluorescence microscopy, and their cross-sectional meniscus profile was determined by image processing. The meniscus of the drops remained circular despite varying concentration. Since the refractive index of corn syrup increased with concentration, while the surface tension coefficient between corn syrup and mineral oil remained unchanged, the observed change in the annulus width was then attributed to the refraction of light passing through the drop’s meniscus. As such, a ray optics model was developed, which predicted that the annulus width of the drop would decrease as the refractive index of the drop approached that of the surrounding liquid. Therefore, the dark annulus of the drops in the Hele-Shaw cell was caused by the refraction of light passing through the circular meniscus of the drop.

## 1. Introduction

**Figure 1:**
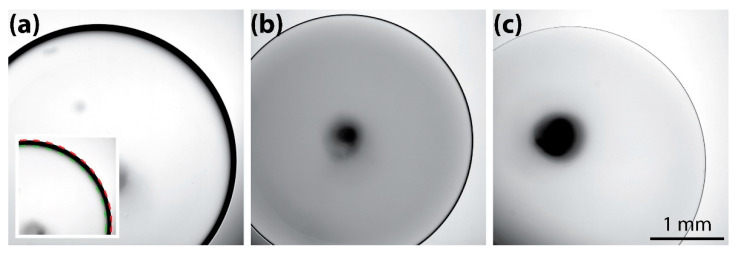
Top-view images of corn syrup drops in a 1 mm thick Hele-Shaw cell filled with mineral oil. The concentration, diameter, and dark ring width of the drops were (**a**) 18% *w*/*w*, 3.73 mm, and 103 µm, (**b**) 67% *w*/*w*, 3.43 mm, and 37 µm, (**c**) 82% *w*/*w*, 3.70 mm, and 9 µm. Inset: circles fitted against the outer (red) and inner (green) boundaries of the dark annulus of the drop.

The presented study began with an intriguing observation that the dark annulus of a disc-shaped drop, which was formed in a Hele-Shaw cell filled with an immiscible liquid, changed in width as the concentration of the drop varied. To investigate the coalescence of drops in the Hele-Shaw cell, we filled a straight microchannel with mineral oil, generated drops of corn syrup, and captured top-view images of the drops using brightfield microscopy. In the images, the drop had a dark ring, or annulus, along its interface with the ambient liquid. As the concentration of corn syrup increased, the width of the dark annulus decreased, as shown in Figure 1.

Previously, we proposed determining the contact angle between a liquid drop and a solid surface by measuring the width of the dark annulus of the drop formed in air between two parallel surfaces of the solid with a small gap (i.e., Hele-Shaw cell) [1]. In our previous study, we found that the width of the dark annulus increased with the contact angle, which was presumably because the meniscus profile of the drop changed as the contact angle changed. The result of the previous study led us to think that the width change in the dark annulus, seen in Figure 1, was due to changes in the meniscus profile of the drop. However, it was noted that the drop meniscus in Figure 1 might not have a contact line because the drop was formed in oil that filled the Hele-Shaw cell. Thus, it could be misleading to guess the cross-sectional profile of the drop meniscus based on the dark annulus.

Knowing the meniscus shape of the drops in the Hele-Shaw cell is important for understanding the coalescence of those drops. For instance, Yokota and Okumura showed that when two drops coalesced in a Hele-Shaw cell, the liquid neck connecting the drops initially grew radially outward until the diameter of the neck became comparable to the gap height of the Hele-Shaw cell [2,3]. Then, this three-dimensional (3D) neck growth became a two-dimensional (2D) neck growth: the neck grew in the spanwise direction of the Hele-Shaw cell because the width of the neck became larger than the gap height. The observed dimensional crossover in the drop coalescence occurring in the Hele-Shaw cell is thought to be affected by the meniscus shape of the drops. When the drop meniscus is convex, the initial contact between two drops occurs near the equator of the drops. If the meniscus is concave (e.g., catenoid-shaped water drops between two hydrophilic surfaces), the initial contact would occur near the contact line. If the meniscus is flat, the coalescence dynamics can be different from one with curved menisci. Therefore, it is important to know the meniscus shape of liquid drops for predicting how they will make an initial contact and then merge in Hele-Shaw cells. 

Confocal fluorescence microscopy (CFM) has been used to image the 3D shape of a meniscus between two immiscible fluids and to obtain a cross-sectional profile of the meniscus [4,5,6,7,8,9,10,11,12,13,14]. CFM yields clear thin optical sections by reducing blurring in the image from out-of-focus light scattering, and the obtained optical sections can be assembled to reconstruct a 3D image. As such, CFM is well suited for imaging the interface, or meniscus, of a liquid drop with immiscible media. For instance, Sundberg et al. and Salim et al. used CFM to obtain the 3D profile of a liquid drop on a solid surface and then to measure the contact angle of the drop [7,8]. Similarly, Kilmametov et al. could measure the height and volume of a spherical cap of a liquid filling a cylindrical cavity using CFM [10]. Recently, Tress et al. and Singh et al. characterized the shape evolution of an evaporating liquid film or meniscus using CFM [11,13]. 

In this study, we imaged corn syrup drops of various concentrations in a Hele-Shaw cell filled with mineral oil using CFM. Note that these liquids are immiscible. Because of the small gap height of the Hele-Shaw cell, it was very challenging to directly observe the meniscus shape from side-view imaging. Instead, a fluorescent dye was added to corn syrup, and its drop was imaged using CFM, which enabled reconstructing a 3D image of the drop meniscus. Then the obtained 3D image was processed to determine the thickness and meniscus profile of the drop based on the cross-sectional images of the drop. The width of the dark annulus was measured from brightfield images of the drops. In the end, a ray optics model was developed to understand why the annulus width changed as the drop concentration changed.

## 2. Materials and Methods

### 2.1. Measurement of Surface Tension Coefficient and Refractive Index

Corn syrup (Light Corn Syrup, Karo) was used as the drop liquid and mineral oil (Food Grade Mineral Oil, Bluewater Chemgroup) as the ambient liquid filling the Hele-Shaw cell. Corn syrup was diluted by mixing it with deionized water (diH_2_O) at weight/weight percentage concentrations (*c*) of 18%, 67%, and 82%. Here, *c* = 100% *w*/*w* means intact corn syrup without any dilution. For CFM imaging, a fluorescent dye (0.1% *w*/*v* of fluorescein sodium salt solution, 46960-100G-F, Sigma-Aldrich, St. Louis, MO, USA) was added to corn syrup solutions with a concentration of 4.8% *v/v* in a centrifugal tube covered by aluminum foil. After the tube was shaken on a mixer for complete mixing of the dye and corn syrup, it stood still overnight to remove air bubbles from the solution. 

The surface tension coefficient (*γ*) between corn syrup and mineral oil was measured using a goniometer (Attension Theta, Biolin Scientific, Gothenburg, Sweden). A cuvette filled with mineral oil was placed on the stage of the goniometer and corn syrup was slowly injected into the oil through a needle by using a syringe pump, to form a pendant drop of a desired size suspending at the needle tip. Upon reaching a steady shape, the drop was imaged for 10 s and *γ* was determined based on the shape of the drop. The *γ* measurement was repeated five times for each concentration of corn syrup. Measured values are summarized in Table 1 in the format of mean ± standard deviation. It needs to be noted that corn syrup solutions for the *γ* measurement did not include the fluorescent dye. Since the concentration of the dye in the corn syrup was very low, negligible change in *γ* due to the dye was assumed [15]. As a validation, the *γ* of diH_2_O in air was measured to be 70 mN/m with the same procedure, which agreed well with the known value (72.75 mN/m at 20 °C) [16]. 

The refractive index (RI) of the used liquids was measured by using a refractometer (0-95 Bx/1.33-1.54 RI, VWR, Radnor, PA, USA). The RI measurement was repeated three times for each sample, and variance was negligible. As a validation, the RI of calibration fluids (RP-CAL46P and RP-CAL60P, Laxco, Mill Creek, WA, USA) was measured to be 46.6 and 60.4, respectively, which were very close to the nominal RI values (46 and 60) of the calibration fluids. As shown in Table 1, the RI of corn syrup increased with the concentration [17,18,19]. 

The density of the working fluids (*ρ*) was measured by measuring the mass of the fluid with a known volume (500 µL or 1000 µL). Repeated measurements showed negligible variance in measured *ρ* values. 

### 2.2. Fabrication of the Hele-Shaw Cell

The gap height in the Hele-Shaw cell (*h*) was determined based on the following consideration of the Bond number (Bo) to ignore any gravitational effects on the drop meniscus. This condition could be achieved when the Bond number (Bo = Δ*ρgh*^2^/*γ*) was smaller than unity (i.e., Bo < 1). Here, Δ*ρ* is the density difference between corn syrup and mineral oil, and *g* is the acceleration of gravity (9.8 m/s^2^). Since *h* needed to be smaller than 2.4 mm, *h* = 1 mm was selected.

A Hele-Shaw cell was made of polydimethylsiloxane (PDMS) and it consisted of two PDMS layers: a microchannel layer and a thin PDMS layer coated on a glass plate. The microchannel layer was fabricated using soft lithography as follows. A master mold was prepared by attaching a piece of 1 mm thick glass coverslip (18 mm × 45 mm) to a glass slide by using adhesive film. The surface of the mold was cleaned by isopropyl alcohol (IPA) and diH_2_O, and then dried with nitrogen gas. Then, the mold was fixed in a Petri dish. Separately, the base and crosslinking agent of PDMS (Sylgard 184, Dow-Corning, Midland, MI, USA) were mixed with a weight ratio of 10:1. 20–25 g of the PDMS mixture was required to make one channel layer of around 4 mm thickness. Well-mixed PDMS was poured onto the mold, and then it was degassed for at least one hour in a vacuum chamber to remove air bubbles from the PDMS mixture. After degassing, PDMS was cured overnight at 60 °C in an oven. After PDMS was fully cured, the channel layer was cut and peeled off from the mold carefully. In the end, a biopsy punch was used to punch one 0.5 mm diameter hole on the channel layer for liquid injection. 

For the bottom PDMS layer, a glass slide (75 mm × 50 mm) was cleaned in IPA and diH_2_O, and then dried by nitrogen gas. Afterwards, the glass slide was loaded in a spin coater (WS-650MZ-23NPPB, Laurell, North Wales, PA, USA) and 2 g of mixed-and-degassed PDMS was poured onto the glass slide. The glass slide was spun at 1000 rpm for one minute to uniformly spread out PDMS. As a result, a 60 µm thick PDMS layer was formed on the plate [20]. Then, the PDMS layer on the glass slide was cured overnight at 60 °C.

**Figure 2:**
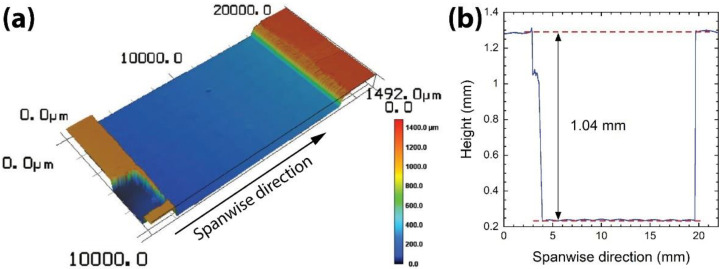
(**a**) The surface topography of the PDMS channel layer of the Hele-Shaw cell. Color bar: height. (**b**) The cross-sectional profile of the PDMS channel layer.

When the two layers were put together, they formed a Hele-Shaw cell of the desired gap height (*h* = 1 mm). The gap height of the Hele-Shaw cell was confirmed by imaging the bottom surface of the PDMS channel layer, which would face the PDMS layer of the glass plate, using a laser scanning confocal microscope (VK-X200, Keyence), as shown in Figure 2a. The tilting angle of the obtained surface image was corrected using the three-point method. The *h* of the Hele-Shaw cell was measured by finding the height difference between the bottom and top surfaces of the channel. As shown in Figure 2b, *h* was measured to be 1.04 mm, which was very close to the desired value (=1 mm). 

### 2.3. Confocal Fluorescence Microscopy Imaging of a Drop

A confocal fluorescence microscope (A1R-Ti2, Nikon, Melville, NY, USA) was used for imaging the drop. A transparent container filled with mineral oil was placed on the stage of the microscope. The Hele-Shaw cell was submerged in the oil and then fixed by placing weights on it. Then, the corn syrup solution with the fluorescent dye was injected through the injection hole of the Hele-Shaw cell using a syringe and tubing. By pushing the plunger of the syringe manually, a corn syrup drop was grown in the Hele-Shaw cell slowly. The injection was stopped when the drop had grown to an approximate diameter of 3.5 mm, and then the device stood still for two minutes to stabilize the drop. 

The drop was imaged by using the microscope with a 4× objective lens (Plan Apo λ, NA = 0.2) and a *z*-step size of 20 μm. The excitation and emission wavelengths were 486 nm and 525 nm, respectively. Since the vertical scanning range was 1.1–1.5 mm, which included the entire gap height of the Hele-Shaw cell, 55–70 layers of 2D fluorescent images were obtained. The size of 2D images was 1024 pixel × 1024 pixel, and the pixel size was 3.11 µm/pixel. 2D images were re-constructed into a 3D image using the microscope software (NIS Elements Viewer, Nikon, Melville, NY, USA), and then the 3D image was saved as a gray-scale tiff image file. A bright-field image of the top view of the drop was also captured.

### 2.4. Image Processing

The thickness and cross-sectional images of the drop were obtained by processing the tiff image file of the drop using MATLAB (MathWorks) [21]. First, the axis of symmetry of the drop was identified. The 20–30 2D images of the drop were converted to binary images using “imbinarize” In each binary image, the drop and the ambient oil looked white and black, respectively. Then, the boundary of the drop was traced using “bwboundaries” and a circle was fitted against the identified boundary using Kasa’s circular fitting method [22]. Then the obtained center coordinates were averaged to determine the coordinate of the axis of symmetry of the drop. 

**Figure 3:**
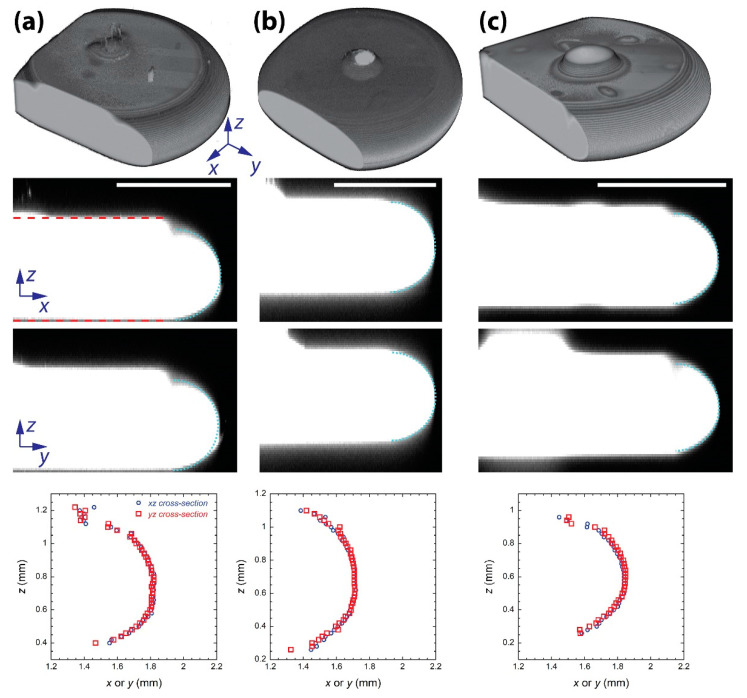
Confocal fluorescence images of the corn syrup drop at a concentration of (**a**) 18%, (**b**) 67%, and (**c**) 82%. Top row: 3D rendered image of the drops. Middle row: *xz*- and *yz*-cross-section of the drop. The left boundary of the images corresponds to the axis of symmetry. Red dashed lines: the top and bottom surface of the drop. Cyan dotted circle: Circle fitted against the identified meniscus of the drop. Scale bar: 1 mm. Bottom row: Comparison of the identified meniscus profiles between the *xz*- and *yz*-cross-sections.

Second, the two cross-sections of the drop were obtained from the reconstructed 3D image with respect to the found axis of symmetry: one for the *xz*-plane and the other for the *yz*-plane (Figure 3). On the basis of the gradient of fluorescence intensity in the *z*-direction, pixels for the maximum gradient magnitude were found in each cross-section, which indicated the drop−oil interface. Since the top and bottom interfaces of the drop were horizontal, the *z*-coordinates of the found pixels were averaged. Then, the thickness of the drop was determined by finding the difference in the *z_avg_* between the top and bottom interfaces.

Last, the meniscus of the drop was identified from each of the cross-sections by finding pixels for the maximum gradient magnitude of fluorescence intensity in the *x*- or *y*-direction. Then, a circular fitting based on Kasa’s method was conducted against the identified meniscus. 

## 3. Results and Discussion

### 3.1. Brightfield Images: Dependence of the Dark Ring Width on Concentration 

Figure 1 shows the brightfield, top-view images of corn syrup drops, with three different concentration (*c*) values (Table 1) in the Hele-Shaw cell with a gap height of *h* = 1 mm. The diameters of the drops (*D*) were around 3.5 mm. The dark annulus can be seen clearly at the interface between the drops and the oil, and the dark annulus became thinner with the increasing concentration of the corn syrup solution.

The width of the dark ring (*w*) was measured as follows. First, the inner and outer perimeter of the dark ring was identified by image processing in a similar way as described in Section 2.4. Second, circles were fitted against the perimeter using Kasa’s method. As shown in the inset of Figure 1a, the fitted circles agreed well with the identified perimeters. Last, the difference in the radius between the circles was determined as the width of the dark annuls. Using this approach, *w* was measured to be 103 µm, 37 µm, and 9 µm for concentrations of 18%, 67%, and 82%, respectively. 

The observed relationship between *w* and *c* is thought to be due to the change in refractive index (RI). The meniscus profile of the drop is determined by a balance between hydrostatic pressure due to density and Young–Laplace pressure due to surface tension (*γ*). Although corn syrup drops had different density as their concentration changed, as shown in Table 1, the density difference is thought to not cause any changes in the meniscus shape because of negligible gravitational effects (i.e., Bo < 1), as discussed in Section 2.2. In addition, *γ* does not show a clear dependence on *c*. Thus, similar meniscus shapes of corn syrup drops are expected, regardless of their concentration, which will be confirmed in the following section. Then, the change in RI appears to be the main reason for the change in *w* because the RI of corn syrup increased with *c*. 

### 3.2. Confocal Images: The Thickness and Meniscus Profile of Drops 

The top row in Figure 3 shows that CFM imaging could successfully capture the 3D shape of the drops, including the part of corn syrup in the injection hole, which appeared as the dark circle in Figure 1. Reconstructed 3D images clearly show that the meniscus of the drops was round, regardless of the concentration of the drop. The round, or even circular, meniscus profile was expected because of the small gap height of the Hele-Shaw cell (i.e., Bo < 1). Similarly, Dolganov et al. found that the cross-sectional meniscus profile of a liquid crystal drop in a Hele-Shaw cell was close to circular [23,24]. 

The middle rows in Figure 3 show the *xz*- and *yz*-cross-sections of the drops, and the red dashed lines indicate the top and bottom surface of the drops that were identified by image processing. The drop thickness measured from the two cross-sections agreed well with each other. For instance, the largest difference in the thickness was found with a drop of 67%: 773 µm from the *yz*-cross-section and 781 µm from the *xz*-cross-section, and this difference was negligible compared to the measured drop thickness. Very close thicknesses between the two perpendicular cross-sections show that the drops were confined between two parallel surfaces, i.e., the Hele-Shaw cell. 

The drop thickness (*t*) was determined by averaging the thickness values obtained from the two cross-sections. *t* was 900 µm, 926 µm, and 777 µm for concentrations of 18%, 67%, and 82%, respectively. It was noted that the measured drop thickness was smaller than the gap height (=1.04 mm). This suggests that mineral oil existed between the drop and the wall of the Hele-Shaw cell, and that the thin oil layer prevented direct contact between the drop and the cell wall. Because of the hydrophobicity and oleophilicity of the PDMS surface of the Hele-Shaw cell, a thin mineral oil layer was expected to exist between the drop and the surface of the Hele-Shaw cell. When an equal thickness was assumed for the top and bottom oil layer, the thickness of the oil layer (*e*) was estimated to be 70 µm, 58 µm, and 132 µm for concentrations of 18%, 67%, and 82%, respectively.

The above oil layer thickness estimation can be improved further by making the PDMS wall of the Hele-Shaw cell visible in CFM imaging. The cell walls are not visible in Figure 3 because they were not fluorescent, which makes it impossible to know the exact thickness of the oil layer and whether the oil layers above and below the drop were similar in thickness. To visualize the Hele-Shaw cell in fluorescence imaging, fluorescent microspheres can be added to PDMS. The emission wavelength of the beads needs to be different from that of the fluorescent dye added to the drop. Adding fluorescent particles in the cell wall will enable identifying the cell wall in the obtained images and, thus, measuring the thickness of the oil layer accurately.

The bottom row of Figure 3 shows that the meniscus profiles of the drop were similar between the two cross-sections. This agreement, along with the negligible difference in the drop thickness between the cross-sections, supports that the drop was axisymmetric. In these graphs, the *x*- or *y*-coordinate values were found with respect to the axis of symmetry of the drop. Thus, the maximum of these coordinates corresponds to the radius of the drop, which agrees well with the drop diameter shown in Figure 1. In contrast, the *z*-coordinate values were found with respect to an arbitrary reference. As such, the graphs were plotted so that the range of the horizontal and vertical axis would be 1 mm, which corresponds to the gap height of the Hele-Shaw cell. It should be noted that the difference in the *z*-values between the highest and lowest points was smaller than 1 mm.

Good agreement between the fitted circle (cyan dotted circles in Figure 3) and the drop meniscus reveals that the meniscus profiles of the drops were circular, regardless of their concentration. This supports our prediction of similar meniscus profiles among the drops, which was made in Section 3.1, based on similar *γ* between the drops and negligible effect of gravity. Then, the variation in *w* is highly likely due to the difference in the RI between the drops. 

It was noted that the meniscus of the drop was not symmetric with respect to its equator. In the middle and bottom rows of Figure 3a,c, the cross-sectional images of the drop show a dimple at the top end of the meniscus. Such dimples are shown as a step-like feature in the 3D image of the drops. It is unclear why the dimpled profile happened on the meniscus. Due to the existence of the dimple, the estimated diameter of the meniscus was smaller than the thickness of the drop. The circle fitting measured that the approximate diameter of the meniscus was 793 µm, 867 µm, and 676 µm for concentrations of 18%, 67%, and 82%, respectively. These meniscus diameters correspond to 88%, 94%, and 87% of the respective drop thickness. 

### 3.3. Ray Optics Model 

**Figure 4:**
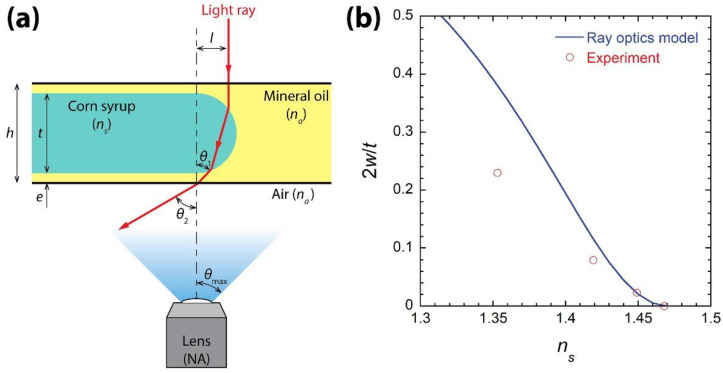
The ray optics model to predict the width of the dark annulus (*w*) of the drop. (**a**) Schematic of the model. (**b**) Comparison of the annulus width normalized by the half thickness of the drop (*t*/2) between the prediction based on the model and the experimental measurement.

As aforementioned, the observed difference in *w* despite the similar circular profiles of the meniscus suggests that the difference was caused not by differenct meniscus profiles but mainly by the different refractive indices of the drops. To test this hypothesis, a ray optics model was developed and *w* was predicted using the model, as shown in Figure 4.

In this model, the meniscus of the drop is assumed to be a semi-circle with a radius of the half thickness of the drop (=*t*/2). Parallel light rays are formed by the condenser of the microscope, and one of them is shown in Figure 4a. The distance between the shown light ray and the center of the circular meniscus is *l*. As this light ray passes through the meniscus, it is refracted two times, as illustrated in Figure 4a. The incidence angle of the shown light ray into the bottom of the Hele-Shaw cell can be assumed as follows [25]:

(1) $$\theta_{1}=2\left(\cos^{-1}\frac{l}{t/2}-\cos^{-1}\frac{l}{n_{s}/n_{o}\cdot t/2}\right),$$

where *n_s_* and *n_o_* are the refractive index of corn syrup and mineral oil, respectively. As the ray enters into air, it is refracted one more time, and the incidence angle of the ray to the lens is

(2) $$\theta_{2}=\frac{n_{o}}{n_{a}}\sin\theta_{1},$$

where *n_a_* is the refractive index of air (=1). Here, refraction through the PDMS and glass of the bottom of the Hele-Shaw cell was ignored for simplicity because the refractive indices of PDMS and glass are close to those of the corn syrups. 

Then, it is assumed that the dark annulus of the meniscus is formed when light rays passing through the ring are not captured by the objective lens of the microscope. Since the numerical aperture (NA) of the lens is the maximum incidence angle of the observable light ray to the lens [i.e., *θ*_max_ = sin^−1^(NA/*n_a_*)] [26], the light ray cannot be captured by the microscope when *θ*_2_ > *θ*_max_. Thus, the part of the meniscus with *θ*_2_ > *θ*_max_ appears dark in a top-view image of the drop. Since *l*_max_ is obtained when *θ*_2_ = *θ*_max_, *l*_max_ is found to be

(3) $$\frac{l_{\max}}{t/2}=\sqrt{\frac{1-C^{2}}{1-\frac{2C}{n_{s}/n_{o}}+\frac{1}{{\left(n_{s}/n_{o}\right)}^{2}}}},$$

where $C=\cos\left[\frac{1}{2}\sin^{-1}\left(\frac{\mathrm{NA}}{n_{o}}\right)\right]$. Therefore, the model enables estimating *w* = *t*/2 − *l*_max_ using Equation (3), the NA of the used objective lens, and the refractive indices of the drop and ambient liquid. 

**Figure 5:**
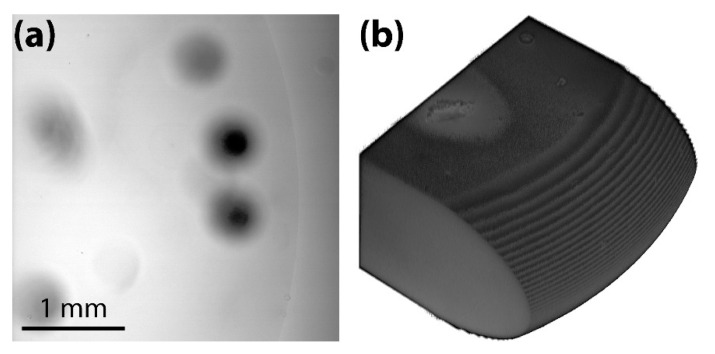
A corn syrup drop with a concentration of 95% *w*/*w* (*n_s_* = 1.468). (**a**) Brightfield, top-view image. (**b**) 3D confocal image (imaged at 10× with NA 0.3).

The width of the dark annulus was calculated using Equation (3) and then normalized with the half thickness of the drop (i.e., 2*w*/*t*), as shown in Figure 4b. The model predicts that *w* decreases as *n_s_* approaches *n_o_*, which agrees with Figure 1. Experimentally obtained 2*w*/*t* values are compared with the model prediction in Figure 4b. The model overpredicted *w* when *n_s_* was much lower than *n_o_*, but the difference between the model and the experimental result decreased as *n_s_* approached *n_o_*. As such, the model agreed with the experiment very well when *n_s_* was 1.445 and 1.468. Here, the case of *n_s_* = 1.468 is described in Figure 5. Therefore, the developed ray optics model can be used for qualitative predictions when *n_s_* differs from *n_o_* and for quantitative predictions when *n_s_* is close to *n_o_*. The agreement between the model and the experiment supports that the observed dependence of *w* on the RI of the drop was mainly caused by the RI difference among the corn syrup drops.

Figure 4b shows that the dark annulus of the drop would become very thin in the top-view image of a drop as *n_s_*/*n_o_* approaches unity (i.e., refractive indices matching). As such, the ray optics model predicted that the dark annulus thickness would be 0 when *n_s_* = 1.47. Such a condition can be achieved by increasing the concentration of corn syrup because Table 1 shows that *n_s_* increased with *c*. Figure 5 shows the brightfield top-view image and confocal 3D image of a corn syrup drop with *c* = 95% *w*/*w*. *n_s_* was measured to be 1.468, which was not only very close to the predicted *n_s_* value, but also was the same as *n_o_*. As predicted by the ray optics model, the dark annulus was invisible in the top-view image (Figure 5a), whereas the drop meniscus was round in the 3D image (Figure 5b). 

## 4. Conclusions

When imaged from the top view using brightfield microscopy, a liquid drop confined in a Hele-Shaw cell appears to have a dark annulus, or ring, along its meniscus interfacing with an ambient immiscible fluid. In this study, it was observed that the dark annulus of corn syrup drops in mineral oil, in the Hele-Shaw cell, became thinner as the concentration of corn syrup increased. This observation was puzzling because the constant surface tension coefficient between the drops and mineral oil and the small Bond number of the drops suggested that the cross-sectional profile of the drop’s meniscus remained unchanged, despite the concentration change. As such, confocal fluorescence microscopy and image processing were used to confirm that the meniscus profiles were circular, regardless of concentration. Furthermore, the developed ray optics model showed that the dark annulus was caused when light rays refracted by the meniscus were not captured by the microscope.

## Figures and Tables

**Table 1:** Properties of the used fluids.

Liquid	Density (g/cm^3^)	Surface Tension Coefficient (mN/m)	Refractive Index (-)
Mineral oil	0.87	-	1.467
Corn syrup (18% *w*/*w*)	1.04	39.2 ± 0.7	1.353
Corn syrup (67% *w*/*w*)	1.26	41.1 ± 1.2	1.421
Corn syrup (82% *w*/*w*)	1.31	40.0 ± 0.3	1.445

## Data Availability

Not applicable.
